# Essential Oils Extracted from Organic Propolis Residues: An Exploratory Analysis of Their Antibacterial and Antioxidant Properties and Volatile Profile

**DOI:** 10.3390/molecules26154694

**Published:** 2021-08-03

**Authors:** Natália Y. Ikeda, Carmen M. S. Ambrosio, Alberto Claudio Miano, Pedro L. Rosalen, Eduardo M. Gloria, Severino M. Alencar

**Affiliations:** 1 Department of Agri-Food Industry, Food and Nutrition, “Luiz de Queiroz” College of Agriculture, University of São Paulo, Piracicaba, São Paulo 13418-900, SP, Brazil; yumi_ikeda@alumni.usp.br; 2 Dirección de Investigación y Desarrollo, Universidad Privada del Norte (UPN), Trujillo 13011, Peru; carmen.sinche@upn.edu.pe (C.M.S.A.); alberto.miano@upn.edu.pe (A.C.M.); 3 Biological Sciences Graduate Program, Federal University of Alfenas—UNIFAL-MG, Rua Gabriel Monteiro da Silva, 700, Alfenas 37130-001, MG, Brazil; pedro.rosalen@unifal-mg.edu.br; 4 Department of Biosciences, Piracicaba Dental School, University of Campinas, Av. Limeira, 901, Piracicaba 13414-903, SP, Brazil; 5 Department of Biological Science, “Luiz de Queiroz” College of Agriculture, University of São Paulo, Piracicaba, São Paulo 13418-900, SP, Brazil

**Keywords:** *Escherichia coli*, HS-GC/MS, *Lactobacillus*, pig production, feedstuff, bee product

## Abstract

The industrial processing of crude propolis generates residues. Essential oils (EOs) from propolis residues could be a potential source of natural bioactive compounds to replace antibiotics and synthetic antioxidants in pig production. In this study, we determined the antibacterial/antioxidant activity of EOs from crude organic propolis (EOP) and from propolis residues, moist residue (EOMR), and dried residue (EODR), and further elucidated their chemical composition. The EOs were extracted by hydrodistillation, and their volatile profile was tentatively identified by GC-MS. All EOs had an antibacterial effect on *Escherichia coli* and *Lactobacillus plantarum* as they caused disturbances on the growth kinetics of both bacteria. However, EODR had more selective antibacterial activity, as it caused a higher reduction in the maximal culture density (*D*) of *E. coli* (86.7%) than *L. plantarum* (46.9%). EODR exhibited mild antioxidant activity, whereas EOMR showed the highest antioxidant activity (ABTS = 0.90 μmol TE/mg, FRAP = 463.97 μmol Fe^2+^/mg) and phenolic content (58.41 mg GAE/g). Each EO had a different chemical composition, but α-pinene and β-pinene were the major compounds detected in the samples. Interestingly, specific minor compounds were detected in a higher relative amount in EOMR and EODR as compared to EOP. Therefore, these minor compounds are most likely responsible for the biological properties of EODR and EOMR. Collectively, our findings suggest that the EOs from propolis residues could be resourcefully used as natural antibacterial/antioxidant additives in pig production.

## 1. Introduction

To protect the hive, *Apis mellifera* L. bees produce a resinous type product named propolis [1,2,3], which is mainly composed of resin (60%) and, to a lesser extent, wax, essential oils, and other constituents [4]. Propolis samples have antibacterial, antifungal, antioxidant, and anti-inflammatory properties [3,5,6] as a result of a complex chemical composition that is highly correlated with the plant species visited by the bees, including the occurrence of volatile compounds [4,7]. While different types of propolis have been extensively known for their polyphenol-rich composition, aromatic compounds may also occur in their chemical profile. The aroma of the sample is relevant for a full characterization of the propolis [4,8]. The ethanolic extract of propolis (EEP) is the main commercialized product, whose industrial preparation yields a resinous residual material [9,10]. Interestingly, propolis residues may harbor underexplored biological activities, such as antimicrobial and antioxidant, which could be resourcefully used for different applications, such as alternative candidates to replace synthetic antibiotics in livestock production.

Antibiotics have been widely used in pig production and other livestock with growth-promoting effects since 1950. This has become a common practice to boost the productivity of livestock production [11,12]. Nonetheless, the overuse of antibiotics for this purpose was recognized as one of the major causes of the emergence and spread of antibiotic-resistant bacteria [13]. The bacterial resistance issue not only affects pig production but can severely compromise human health through the transfer of resistant strains and their associated genes via the food chain [13]. To overcome this public health issue, restrictions on the use of antibiotics in livestock were adopted in 2006 by the European Union under regulation No. 1831/2003 [14]. Currently, other countries around the globe, such as Japan, China, Canada, United States, and Brazil, have also adopted measures to regulate the use of some antibiotics in livestock production [15,16,17,18], which ultimately encourages the search for alternative candidates to replace standard drugs.

Over the years, essential oils (EOs) have been regarded as a promising natural alternative to antibiotics, particularly in pig production, due to their ability to improve the growth performance of pigs at levels similar to those of antibiotics [19]. The growth-promoting effects of EOs were associated with their benefits to gut health via stimulation of endogenous digestive secretion (e.g., enzymes, bile, and mucus), maintenance of the intestinal epithelial structure, and modulation of the gut microbiota [20,21]. This last effect is mainly reached by the antimicrobial activity of EOs on the pig gut microbiota in a way that EOs suppress or reduce pathogenic bacteria while not affect or have a low effect on beneficial bacteria of the pig gut [21,22]. EOs from propolis have already been shown to have an antibacterial effect on several Gram-negative and Gram-positive pathogenic bacteria such as *Escherichia coli, Enterobacter cloacae, Klebsiella pneumonie, Pseudomonas aeruginosa, Staphylococcus aureus, Enterococcus faecalis, Streptococcus pyogenes, Streptococcus mutans* [4,23].

In addition to antibiotics, synthetic antioxidants are also commonly used as an effective feed additive in pig diets to increase the stability of the feedstuff by protecting nutrients (e.g., fat and vitamins) from oxidation and decreasing the oxidative stress in animal tissues, such as the intestinal mucosa. However, the use of synthetic antioxidants has been questioned due to their potential adverse effects on the pigs’ health [24]. This problematic situation has driven the search for natural compounds that could not only replace synthetic antioxidants but also provide additional zootechnical benefits. As a potential solution, EOs have been reported to efficiently scavenge free radicals [21] and thus could be considered an excellent choice to replace both antibiotics and synthetic antioxidants.

The incorporation of EOs from propolis residues into pig feedstuff could supply bioactive compounds at a low cost with antimicrobial and antioxidant properties while contributing to waste reduction and environmental protection [24,25]. In this study, the EOs from crude propolis (EOP) and from its moist residue (EOMR) and dry residue (EODR) were tested for their in vitro antibacterial on *Escherichia coli* ATCC 25922 (pathogenic model) and *Lactobacillus plantarum* ATTC 8014 (beneficial model), which are microorganisms commonly occurring in the pigs’ gut. Next, the EOs were further tested for their antioxidant properties and characterized for their volatile composition.

## 2. Materials and Methods

### 2.1. Crude Propolis Samples and Industrial Residues

**Figure 1:**
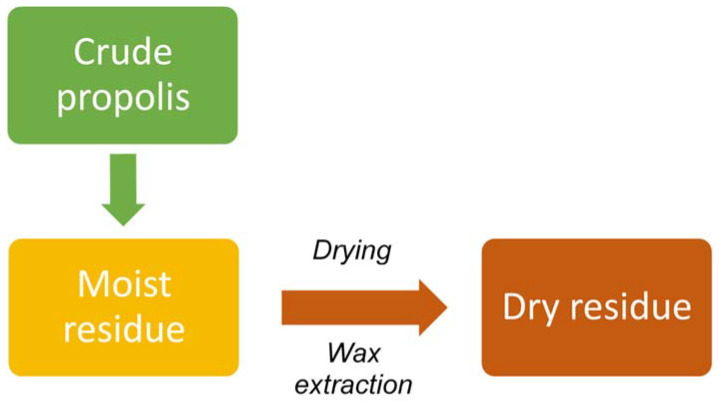
Flowchart of the processing to obtain propolis residues.

Crude organic propolis and industrial residues from the ethanolic extraction of propolis were provided by *Breyer—Naturais e Orgânicos* (União da Vitória, Paraná State, Brazil, 26°11′50.4″ S, 51°06′49.9″ W). Crude organic propolis was obtained in pieces, whereas the moist propolis residue was obtained in a solid form soaked in ethanol and the dry residue was provided in dried form (Figure 1). The moist residue was first dried at room temperature and then in an oven at 60 °C to eliminate all the solvent.

### 2.2. Essential Oil Extraction

The essential oils from crude propolis (EOP) and from its moist residue (EOMR) and dry residue (EODR) were obtained by hydrodistillation for 4 h in a Clevenger-type apparatus, as described by [26]. The samples were five-fold diluted in distilled water for distillation. The volume of the EOs was measured to calculate the extraction yield. The samples were stored in amber flasks at 4 °C until use.

### 2.3. Chemical Volatile Profile

The chemical composition of the EOs was determined by Headspace Gas Chromatography/Mass Spectrometry (HS-GC/MS) in a GCMS-QP2010 Plus (Shimadzu Corp., Tokyo, Japan). The samples were placed in vials and heated at 40 °C with agitation for 5 min in a heating module to release the volatile constituents. After heating, 500 μL of the gaseous phase were collected and injected at a 1:50 split ratio. The separation of volatile compounds was performed on an Rtx^®^-5 MS column (5% diphenyl/95% dimethyl polysiloxane 30 m × 0.25 mm ID × 0.25 μm film thickness) (RESTEK). The temperature ramp began at 40 °C and was maintained for 4 min, followed by 150 °C at 3 °C/min and 250 °C at 15 °C/min, maintained for 2 min. Helium was used as a carrier gas at a linear velocity of 36.1 cm/s. Mass spectra and total ion currents (TIC chromatograms) were obtained by automatic scanning with energy ionization at 70 eV in the mass range 35–500 *m*/*z*. To tentatively identify the chemical constituents, calculated retention indices (RI_calc_) were compared to the literature (RI_lit_). Mass spectra were compared to both the published literature [27] and MS libraries (Wiley^®^ 8, New York, NY, USA and FFNSC 1.3).

### 2.4. Antibacterial Activity

#### 2.4.1. Bacterial Strains and Growth Conditions

The following bacterial strains from the American Type Culture Collection (ATTC, Rockville, MD, USA) were used: *Escherichia coli* ATCC 25922—as a pathogenic model—and *Lactobacillus plantarum* (ATCC 8014)—as a beneficial model. *E. coli* was cultivated in Tryptic Soy Agar (TSA, Difco^TM^, France) at 37 °C for 18–20 h and *L. plantarum* in De Man, Rogosa, and Sharpe agar (MRS, Difco^TM^, France) at 30 °C for 48 h. After activation, the strains were sub-cultured in Brain-Heart Infusion (Difco^TM^, France) or MRS broth supplemented with 15% of glycerol and stored at −20 °C until use.

#### 2.4.2. Determination of the Minimum Inhibitory Concentration (MIC)

The Minimum Inhibitory Concentration (MIC) of the EOs was determined by the broth microdilution method based on the Clinical and Laboratory Standards Institute guidelines (M07-A9) [28]. The assays were performed in a 96-well microplate containing Mueller-Hinton (MH) broth (*E. coli*) or MRS broth (*L. plantarum*). In total, 50 μL of the EOs were dissolved in 25 μL of Tween 80 (emulsifier) and MH or MRS broth was added to obtain a stock solution of 1500 μL. The EO stock solution was added to the wells and serially diluted (1:2 ratio) from 14.8 to 0.12 mg/mL.

Living colonies from MH (*E. coli*) or MRS (*L. plantarum*) agar plates were suspended in saline solution (0.85% NaCl, *v*/*v)* to an optical density of 10^8^ CFU/mL (0.08–0.13 at 625 nm). Subsequently, the inoculum was diluted at 1:100 to obtain a final concentration of 10^6^ CFU/mL (final inoculum). Then, 20 μL from the final inoculum were added to each well of the microplate with 180 µL of the serially diluted EO sample, totaling a final volume per well of 200 µL and a final cell count of 10^5^ CFU/mL. A Tween 80 stock solution (25 μL of Tween 80 complemented with MH or MRS broth to a final volume of 1500 μL) was used as a control. The plates were incubated in a microplate reader incubator (Vitor^TM^ X3, PerkinElmer) at 35 °C for 24 h (*E. coli*) or 30 °C for 36 h (*L. plantarum*). The existence or not of bacterial growth was evaluated by optical density measurements at 600 nm (OD_600_). The lowest concentration of the EOs that did not produce detectable OD_600_ until the end of the incubation period was considered the MIC. All assays were carried out in triplicate in three independent experiments.

#### 2.4.3. Bacterial Growth Modeling and Calculation of Kinetics Parameters

The growth kinetics of EO-treated bacterial cultures were measured hourly for 24 h (*E. coli*) or 36 h (*L. plantarum*) at OD_600_. The Gompertz model modified by [29] Equation (1) was used to adjust the data, as follows:

(1) $$y=D.\exp\left\{-exp\left[\frac{{µ}_{\max}.e}{D}\left(\lambda -t\right)+1\right]\right\}$$

where *y* represents the relative population size against time; *D* represents the maximal bacterial culture density at 600 nm; µ_max_ corresponds to the maximum specific growth rate (h^−1^); λ is the lag phase duration (h). Model parameters for each treatment were obtained by non-linear regression. For this, a Levenberg–Marquardt algorithm was used in STATISTICA 12.0 (StatSoft, Inc., Tulsa, OK, USA). The Mean Square Error (MSE) and corrected determination coefficient (R^2^) for each set of data were used to ensure the suitability of the adjustments to the model.

### 2.5. Antioxidant Activity

Prior to the antioxidant assays, EO samples were diluted in ethanol (P.A.) and homogenized for 45 min in an ultrasound water bath at 45 °C.

#### 2.5.1. ABTS Free Radical Scavenging Assay

The antioxidant capacity of the EOs was determined by the free radical ABTS (2,2′-azino-bis-3-ethylbenzothiazoline-6-sulphonic acid) assay, according to [30] with modifications. The radical ABTS stock solution was diluted in 75 mM potassium phosphate buffer (pH 7.4) and stored at room temperature for 16 h. The solution was then diluted in ethanol (P.A.) to an optical density of 0.700 ± 0.020 at 734 nm. Aliquots of 30 μL of the EOs and EEP diluted in ethanol were added to 3 mL of the ABTS radical solution and kept in the dark at room temperature. The optical density was measured after 6 min of the beginning of oxidation. Ethanol (P.A.) was used as a blank and Trolox was used as a standard at concentrations ranging from 1000 to 62.5 μM. The optical density was measured at 734 nm, and the results were expressed as μmol of Trolox equivalents (TE) per mg of the sample (μmol TE/mg).

#### 2.5.2. DPPH Free Radical Scavenging Assay

The DPPH (2,2-diphenyl-1-picryl-hydrazyl-hydrate) free scavenging activity of the EOs was determined as previously described [31]. The reaction mixture consisted of 500 μL of the diluted solutions of EOs and EEP, 3 mL of ethanol, and 300 μL of a 150 μM DPPH radical solution in ethanol (P.A.). After 45 min in the dark, the optical density was measured at 517 nm. Ethanol was used as a blank and a calibration curve was built with Trolox as a standard at concentrations ranging from 10 to 100 μM. The results were expressed as μmol of Trolox equivalents (TE) per mg of sample (μmol TE/mg).

#### 2.5.3. Ferric Reducing Antioxidant Power

The analysis consists of the reduction of Fe^3+^ with 2,4,6-tris(2-pyridyl)-s-triazine (TPTZ) in an acid reaction condition, as previously described [32]. Fe^3+^ reduction to Fe^2+^ in a complex with TPTZ increases the optical density at 595 nm. The FRAP reagent was prepared with 50 mL of buffer acetate (300 mM, pH 3.6), 5 mL of TPTZ solution (10 mM TPTZ in 40 mM HCl), and 5 mL of FeCl_3_ (20 mM) in an aqueous solution. Aliquots of 120 μL of the EOs and EEP were added with 180 μL of distilled water and 1.2 mL of FRAP reagent. The optical density was measured at 595 nm after 8 min of incubation at 37 °C. Distilled water was used as a blank, and a calibration curve was plotted using ferrous sulfate as a standard. The results were expressed as μM of Fe^2+^ equivalents per mg of sample (μM Fe^2+^/mg).

### 2.6. Total Phenolic Content

The total polyphenol content of the EOs was determined by the Folin–Ciocalteu spectrophotometer method, as previously described [33]. The EOs and EEP were diluted in ethanol (P.A.) and subjected to the same homogenization procedures described in Section 2.5. A 150-μL aliquot of the EOs was mixed with 750 μL of Folin–Ciocalteu reagent (1:10) and 600 μL of 7.5% Na_2_CO_3_. The optical density was measured in a spectrophotometer UV-mini 1240 (Shimadzu-Co) at 740 nm after 2 h of incubation at room temperature in the dark. Distilled water under similar conditions served as a blank, and a calibration curve was plotted using gallic acid as a standard. The results were expressed as mg of gallic acid equivalents per g of sample (mg GAE/g).

### 2.7. Statistical Analysis

All assays were performed in triplicate and the results were expressed as means ± standard deviation. Analysis of variance (ANOVA) followed by Tukey’s post hoc test was used to detect significant differences between the EO treatments in the growth kinetics parameters (*D*, µ_max,_ and λ) and antioxidant activity. The chemical composition of the samples was analyzed by principal component analysis (PCA). The data were analyzed in the R software and were considered statistically significant at *p* < 0.05.

## 3. Results

### 3.1. Extract Yield and Antibacterial Activity of the EOs

The extraction yield of the EOs was 1.13% for crude propolis (EOP), 0.12% for the moist residue (EOMR), and 0.16% for the dry residue (EODR). The ~10-fold lower yield of the EOs extracted from propolis residues as compared to that of the crude samples indicates that the processing of propolis for ethanolic extract production removed most of the EO constituents present in crude propolis.

**Figure 2:**
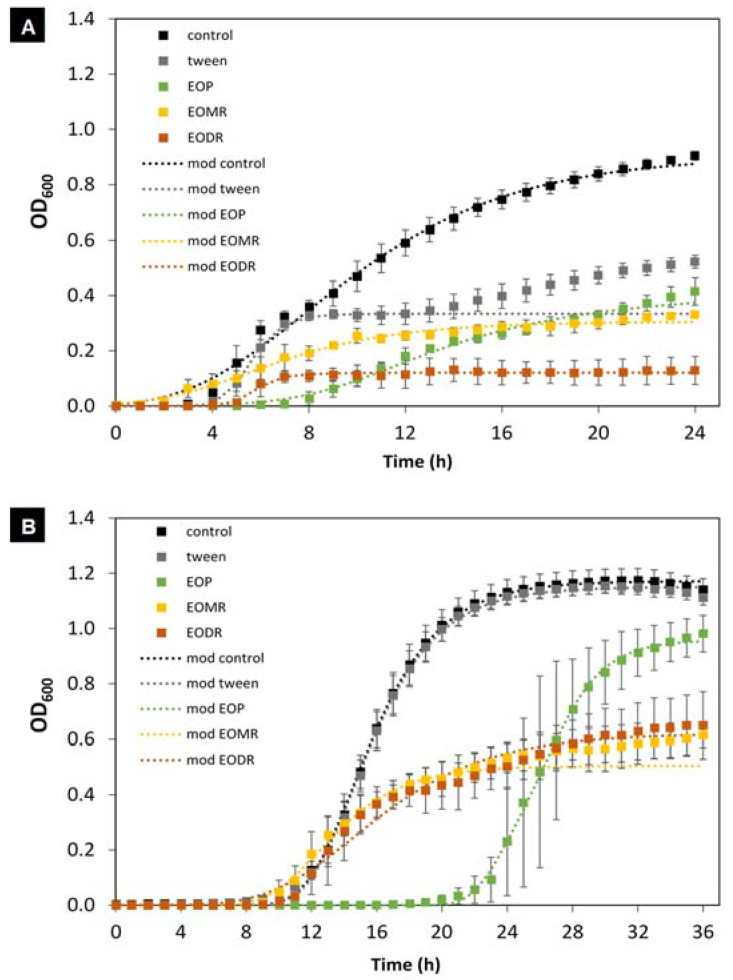
The effects of essential oils from crude organic propolis (EOP) and from its moist residue (EOMR) and dry residue (EODR) on the growth kinetics of *E. coli* (**A**) and *L. plantarum* (**B**) at the concentration of 14.8 mg/mL. Dotted curves indicate the experimental data and dashed curves are the adjusted data using the modified Gompertz model. Vertical bars represent the standard deviation.

**Figure 3:**
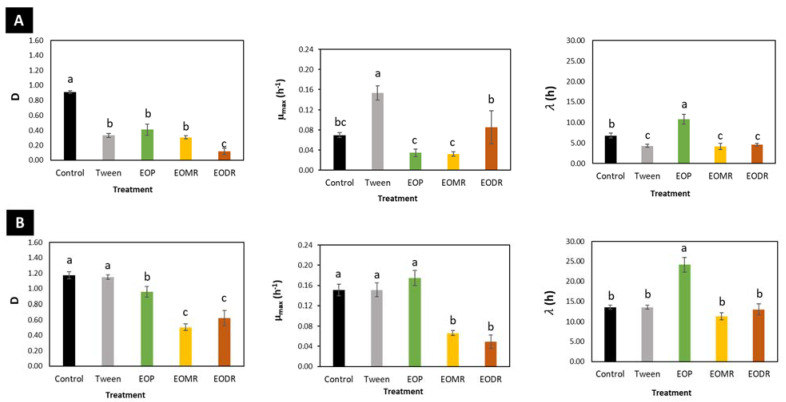
The effects of essential oils from crude organic propolis (EOP) and from its moist residue (EOMR) and dry residue (EODR) on the maximal bacterial culture density (*D*), maximum specific growth rate (µ_max_), and lag phase duration (λ) of *E. coli* (**A**) and *L. plantarum* (**B**). Columns with different letters indicate statistically significant differences (*p <* 0.05). Vertical bars represent the standard deviation.

The antibacterial effects of the EOs on *E. coli* and *L. plantarum* are shown in Figure 2 and Figure 3. The highest tested concentration of the EOs (14.8 mg/mL) significantly affected the growth kinetics of *E. coli* and *L. plantarum* (Figure 2). Both bacteria were susceptible to the effects of the EOs as treated cultures showed lower optical density at the end of the incubation time compared to that of the untreated control (without EO). However, the antibacterial effects of the EOs (EOP, EODR, and EOMR) were stronger on *E. coli* than *L. plantarum*. The modified Gompertz model (Equation (1)) was used to fit the bacterial growth data, and the adjusted data depicted the activity of the samples (EOP, EOMR, and EODR) until the maximum Log phase. Thus, this model allowed the evaluation of maximal bacterial culture density (*D*), maximum specific growth rate (µ_max_), and lag phase duration (λ). The parameter values for each treatment group are shown in (Figure 3).

Tween 80, which was used to emulsify the EOs stock solution, had some antibacterial activity on *E. coli*, as significantly reduced *D* (63.1%) and λ (36.8%) and increased µ_max_ (120%). However, it did not have any antibacterial effects on *L. plantarum*.

Treatment with EODR significantly affected the growth kinetics of *E. coli*, with a significant reduction in *D* (86.7%) compared to the control (*p* < 0.05), whereas EOP and EOMR had effects like those of Tween 80 on this parameter. The EOs from propolis and its residues significantly decreased the parameter *D* of *L. plantarum* (*p* < 0.05), particularly, EOMR (56.9%) and EODR (46.9%) caused a larger reduction compared to EOP (18.2%).

In *E. coli*, EOP and EOMR were able to reduce the maximal growth rate (µ_max_) to 0.034 h^−1^ (50.5%) and 0.032 h^−1^ (54.1%), respectively, although this was not significant compared to the control (0.069 h^−1^). In contrast, treatment with EODR increased slightly, albeit non-significantly, the µ_max_ (0.085 h^−1^) of *E. coli*. In *L. plantarum*, EOMR and EODR significantly (*p* < 0.05) reduced the µ_max_ to 0.066 h^−1^ (56.3%) and 0.049 h^−1^ (67.8%), whereas no effect was observed for EOP compared to the control (0.151 h^−1^).

Treatment with EOP significantly increased the parameter λ (*p* < 0.05) by approximately 1.6-fold in *E. coli* and 1.8-fold in *L. plantarum*. In contrast, treatment with EOMR and EODR significantly decreased λ of *E. coli* (*p* < 0.05) similarly to Tween 80, while none of these treatments affected this parameter of *L. plantarum*.

Collectively, our results showed that EODR exhibited the strongest antibacterial activity on *E. coli* growth kinetics as it causes the highest disturbance on the normal growth kinetic of this bacterium. Similarly, EODR and EOMR exhibited the strongest effects on the growth kinetics of *L. plantarum*, although they were less pronounced than those on *E. coli*. Thus, the data suggest that EODR had a selective antibacterial effect, that is, a stronger effect on the pathogenic bacterium model (*E. coli*) and a mild effect on the beneficial bacterium model (*L. plantarum*).

### 3.2. Phenolic Content and Antioxidant Activity

**Figure 4:**
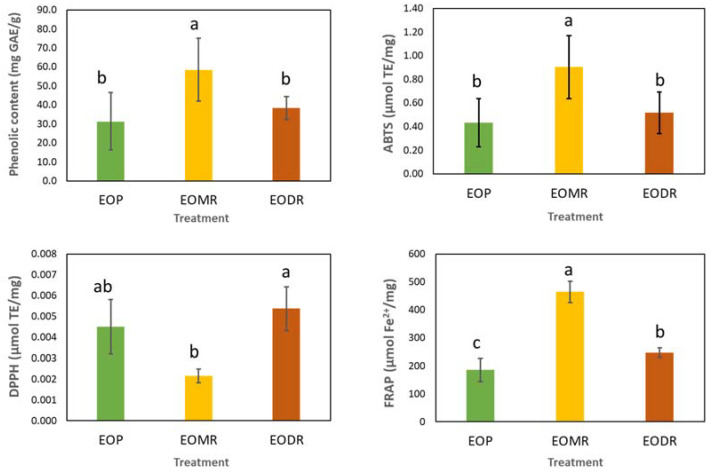
Phenolic content and antioxidant activity [(ABTS and DPPH free radical scavenging and ferric reducing antioxidant power (FRAP)] of essential oils from crude organic propolis (EOP) and from its moist residue (EOMR) and dry residue (EODR). Columns with different letters indicate statistically significant differences (*p <* 0.05). Vertical bars represent the standard deviation.

The results of the total phenolic content analysis and antioxidant activity are shown in Figure 4. Overall, EOMR exhibited the highest phenolic content (58.41 mg GAE/g), followed by EODR (38.31 mg GAE/g) and EOP (31.34 mg GAE/g)—which had a similar content of phenolic compounds. Furthermore, EOMR showed the highest antioxidant capacity in terms of ABTS (0.90 μmol TE/mg) and FRAP (463.97 μmol Fe^2+^/mg) followed by EODR and EOP. Nevertheless, all EOs showed a low capacity to scavenge the DPPH radical, with values ranging from 0.002 to 0.005 μmol TE/mg.

### 3.3. Chemical Composition and Its Relationship with the Biological Activities

**Figure 5:**
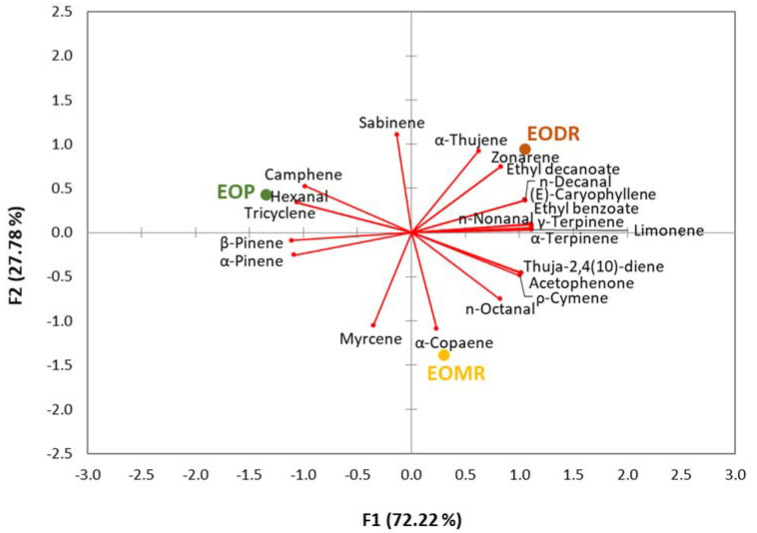
Principal components analysis (PCA) of the chemical composition profile of essential oils from crude organic propolis (EOP) and from its moist residue (EOMR) and dry residue (EODR).

The HS-GC/MS analysis tentatively identified 14 compounds in EOP (97.46%), 16 compounds in EOMR (75.43%), and 18 compounds in EODR (53.95%) (Table 1). As shown in Figure 5, each EO had a different chemical profile. The first and second dimensions of the PCA explained 72.22% and 27.78% of the total variance, respectively. Alpha-pinene and β-pinene were the major compounds detected in EOP, EOMR, and EODR, but the highest relative abundance of both compounds was observed in EOP samples (66.48% and 18.46%, respectively). In addition, the compounds camphene (2.94%), tricyclene (1.54%), and hexanal (0.19%) were highly correlated with EOP (Figure 5, Table 1). EOMR had the highest abundance of thuja-2,4(10)-diene (5.02%), *p*-cymene (2.29%), myrcene (1.32%), acetophenone (0.68%), and *n*-octanal (0.60%), and exclusively α-copaene (0.93%) (Figure 5 and Table 1). EODR had a high correlation with ethyl benzoate (7.8%), limonene (3.07%), (E)-caryophyllene (3.02), *n*-decanal (1.32%), α-thujene (1.13%), γ-terpinene (0.99%), *n*-nonanal (0.97%), α-terpinene (0.91%), sabinene (0.50%), ethyl decanoate (0.74%) and zonarene (3.82%), which were in a higher relative amount in this EO than in EOP or EOMR. Of note, the relative abundance of α-pinene and β-pinene was reduced in the EOs extracted from propolis residues, particularly EODR had the lowest relative amount of both compounds.

Taken altogether, the results of the biological activities suggest that the EOs extracted from propolis residues (EOMR and EODR) exhibited stronger antibacterial and antioxidant activity than the EO extracted from crude propolis (EOP). The EOs from propolis residues were characterized by a higher relative amount of thuja-2,4(10)-diene, *n*-octanal, α-terpinene, *p*-cymene, limonene, γ-terpinene, acetophenone, *n*-nonanal, ethyl benzoate, *n*-decanal, (e)-caryophyllene. These compounds are most likely to be associated with the biological activities observed for these EOs. EODR showed the strongest selective antibacterial activity and exhibited mild antioxidant activity, which may be related to the occurrence of those minor compounds detected in higher amounts in EODR than EOP. Similarly, the strongest antioxidant activity exhibited by EOMR could be associated with the 6 minor compounds detected in a higher relative amount as compared to EODR and EOP samples.

The data were represented as relative quantities for each essential oil; the tentative identification of the compounds present in the samples did not reach 100%.

## 4. Discussion

The extraction of EOs from residues generated upon ethanolic extraction of propolis could increase the sustainability of the propolis chain by adding value to the otherwise-discarded waste while reducing environmental pollution. EOs from propolis residues could have an interesting application in animal nutrition as a natural alternative to synthetic products currently used due to their biological properties, such as antibacterial and antioxidant activity. Moreover, the low cost of propolis residues can substantially minimize the production costs of animal livestock [24,25].

Our results showed that the EOs extracted from organic propolis and its residues had a low extraction yield (0.12–1.13%), which is in line with previous reports for EOs from Brazilian propolis. For instance, [34] obtained a yield of 0.25% during the extraction of the EO from Brazilian red propolis. Similarly, [8] reported a yield of 0.07% for the EO of propolis from the Brazilian Cerrado biome, whereas [26] reported a yield of 0.06% for the EO of propolis from Rio de Janeiro. In our study, the EOP had a higher yield than that of the EOs from propolis residues, probably because raw propolis expectedly harbors a higher concentration of volatile compounds than its residues. The processing of raw propolis generates residues with a low concentration or no traces of volatile compounds [35], particularly the dry residue since it is further subjected to drying and wax extraction.

In our study, the EOs of propolis and its residues were mainly composed of α-pinene, β-pinene, and limonene, although different concentrations were detected in each EO. In addition, nine common compounds were present in all EOs. The processing of propolis samples most likely caused changes in their volatile composition. EOMR was obtained after ethanolic extraction and drying of the moist residue at 60 °C, whereas EODR was produced after the drying procedure and withdrawal of the wax. These biochemical changes in propolis residues are similar to those that occur during the drying of herbs, including alterations in the aroma produced by the loss of volatiles or the formation of new volatiles by oxidative or esterification reactions [36].

The EOs of propolis and its residues analyzed in our study showed a quite similar composition to that of the crude Brazilian green propolis [37], which presented α-pinene and β-pinene as major compounds. However, those authors found that the chemical composition varies depending on the green propolis quality. Similarly, [1] observed that propolis from various localities of South Africa was predominantly composed of α-pinene (1.2–46.5%), β-pinene (2.0–21.8%), limonene (trace-11.6%), 1,8-cineole (0.1–11.0%), and α-thujene (trace-11.0%) when analyzed by solvent-free Head Space technique, the same technique used in our study. The author highlighted that this technique allowed the extraction of volatile constituents present in propolis resins. Likewise, [38] also detected high amounts of α-pinene (20.57–53.41%) and β-pinene (8.86–27.44%) in propolis samples from Brazil, China, Estonia, and Uruguay. Similarly, [39] identified a predominance of α-pinene (57–63%), β-pinene (12.5–30.8%) and limonene (1.5–11.2%) in EOs of propolis from Rio Grande do Sul State (Brazil)—the same three major compounds detected in our study. However, it has been reported that the relative amount of α-pinene and β-pinene in EOs of propolis from different Brazilian regions varies and that these compounds are not always the major constituents [4]. This is the case of the EO from Brazilian red propolis, which presented methyl eugenol (13.1%) as the major compound, followed by (E)-β-farnesene (2.5%) and δ-amorphene (2.3%) [34]. Similarly, the EO of propolis from the Cerrado biome was mainly composed of (E)-caryophyllene (7.85%), δ-cadinene (7.67%), spathulenol (6.65%), viridiflorene (4.52%), α-copaene (4.01%), aromadendrene (3.85%), α-trans-bergamotene (3.73%) and (E)-nerolidol (3.72%) [8]. Likewise, the most abundant compounds in propolis samples from Rio de Janeiro State (Brazil) were β-caryophyllene (12.7%), acetophenone (12.3%), and linalool (6.47%) [26]. Several studies have indicated that the volatile chemical composition of propolis can change with the geographical origin due to variations in the flora, weather pattern, type of bee involved in the pollination process, among other factors [1,4].

Overall, the EOs from propolis and its residues had antibacterial effects on *E. coli* and *L. plantarum* as they caused disturbances on the normal growth kinetics of both bacteria. *E. coli* was more susceptible to the treatment with EODR than *L. plantarum*, suggesting that it had selective antibacterial activity. Selectivity towards pathogenic bacteria rather than beneficial bacteria is a desired feature in the antibacterial spectrum of EOs as a potential alternative to synthetic antibiotics used as growth promoters in pig production. It is intended that EOs modulate the pig’s gut microbiota [21,40], reducing the load of harmful bacteria such as *E. coli* while having a minimal or no effect on beneficial bacteria such as lactobacilli [22,41]. Antimicrobial selectivity is a relevant characteristic [22,42] to promote a healthy gut and, consequently, maintain animal welfare and better performance [43,44]. Just a few studies are reporting on the antibacterial activity of EOs from propolis and most of the literature in the field addresses ethanolic extracts of propolis [4,45]. To the best of our knowledge, this is the first report on the antibacterial activity of EOs extracted from propolis residues. The EO of propolis from Rio Grande do Sul (Brazil) was previously shown to have antibacterial activity on *Staphylococcus aureus* ATCC 6538, *Bacillus subtilis* ATCC 6633, *Pseudomonas aeruginosa* ATCC 25619, *Klebsiella pneumoniae* ATCC 1003, and *E. coli* ATCC 25792. [39]. Other authors also reported that the EO of propolis from Rio Janeiro (Brazil) was effective against several Gram-positive bacteria and even more effective against *E. coli* [26]. Likewise, the EO of propolis samples from different parts of Greece presented a higher antibacterial effect on several Gram-negative bacteria than Gram-positive bacteria, showing the strongest activity on *E. coli* ATCC 25922 at concentrations ranging from 3.4 to 4.9 mg/mL [23]. Thus far, no studies are reporting the effects of EOs from propolis on *Lactobacillus* spp. However, the effects of an ethanolic extract of propolis from different localities of Turkey and Brazil on *L. acidophilus* ATCC 4356 were previously reported by [46]. These authors found that the ethanolic extract of propolis inhibited or killed *L. acidophilus* at concentrations of 4–64 µL/mL and 8–128 µL/mL, respectively.

In our study, while the EOs from propolis (EOP) and its residues (EOMR and EODR) did not completely inhibit the growth of *E. coli* ATCC 25922 and *L. plantarum* ATCC 8414 at the tested concentrations (0.116–14.8 mg/mL), they showed remarkable antimicrobial effects on the growth kinetics parameters of both strains at the highest concentration (14.8 mg/mL). Interestingly, treatment with EODR reduced the maximal bacterial density of *E. coli* (86.7%) more pronouncedly than *L. plantarum* (46.9%). The stronger effects of some EOs or isolated EO compounds on the growth kinetics of *E. coli* than *Lactobacillus* spp. have already been reported. Oregano, thyme, and rosemary EOs, and the isolated EO compounds carvacrol, eugenol, and thymol, at concentrations ranging from 0.005 to 0.5 mg/mL caused a greater reduction of the maximal bacterial density of two *E. coli* (K88^+^) strains isolated from porcine diarrhea as compared to *L. fermentum* and *L. reuteri* [47]. Similarly, a recent study reported that a commercial citrus EO altered to a higher extent the growth kinetics of an enterotoxigenic *E. coli* (ETEC) strain isolated from the pig gut than *L. rhamnosus*. The citrus EO caused a greater reduction of the maximal bacterial density (38.9–55.9%) and higher extension of the lag phase duration (12.2–633.9%) of ETEC than *L. rhamnosus* (Lag phase extended to 16.5–60.2%) at concentrations ranging from 0.116 to 0.925 mg/mL [22]. Compared to these EOs and EO compounds, the EODR presented a lower selective antibacterial activity as it altered the growth kinetics parameters of *E. coli* and *L. plantarum* at a higher concentration.

The literature has shown that. combined EOs can more effectively disrupt the growth kinetics of *E. coli*, specifically the lag phase duration, as compared to the EOs tested individually. For instance, the combination of oregano/basil, oregano/balm, oregano/sage, and oregano/thyme significantly extended the lag phase duration of *E. coli* ATCC 25922 to a higher extent than that of oregano EO alone [48].

Our study showed that EODR reduced the maximal bacterial density (46.9%) and growth rate (to 0.049 h^−1^) of *L. plantarum*. Consistent with this, a previous study showed that *Melaleuca armillaris* EO reduced the maximal bacterial density (51.8–96%) and growth rate (to 0.123–0.037 h^−1^) of *L. plantarum* at concentrations ranging from 0.25 to 25 µg/mL [49]. Thus, EODR exerted a lower effect on the growth kinetics parameters than the *M. armillaris* EO. The antibacterial mechanism of action of the bioactive products occurring in propolis is related to their disruptive effects on the permeability of the bacterial cell membrane, membrane potential, and adenosine triphosphate (ATP) production as well as to a reduction in bacterial motility [50]. The antibacterial activity exhibited by the EOs tested herein is likely to be related to these effects in *E. coli* and *L. plantarum*. The selective antibacterial activity observed for EODR may be associated with its more pronounced effects on *E. coli* cells rather than *L. plantarum* cells.

The antimicrobial activity of propolis products, such as EOs, can be related to their highly complex and variable compounds and the synergistic action between them [51]. Several studies have attributed the antibacterial activity of EOs to their major compounds only. In our study, while two common major compounds were detected (α-pinene, and β-pinene) in EOP, EOMR, and EODR, the samples presented different antibacterial activity. On one hand, the monoterpenic composition seems to contribute to the biological properties of the EO from propolis, including the compounds α-pinene and β-pinene [39]). These compounds have already been proven to have inhibitory effects on *E. coli*, *S. aureus*, *Salmonella enterica*, among others [52,53,54]. On the other hand, previous studies reported weak or no antibacterial activity of α-pinene and β-pinene on several pathogenic and beneficial strains, including *E. coli* and *L. plantarum* [55,56]. Thus, it is possible to infer that these two major compounds are not exclusively responsible for the antibacterial activity of EOP, EOMR, and EODR. Moreover, these EOs presented a different relative amount of α-pinene and β-pinene, whose concentration was reduced throughout the processing of crude propolis to residues. The lower relative amount of α-pinene and β-pinene in EODR may be related to the drying and wax removal process, which likely favored the loss of these compounds. Moreover, while EOP presented the highest amount of α-pinene and β-pinene, EODR showed the highest selective antibacterial activity. Interestingly, EODR presented a higher relative amount of α-thujene, α-terpinene, γ-terpinene, *n*-nonanal, ethyl benzoate, *n*-decanal, (E)-caryophyllene, sabinene, ethyl decanoate, and zonarene. Such minor compounds could be mechanistically related to the selective antibacterial activity of EODR rather than the major compounds only.

The selective antibacterial activity of some of the minor compounds detected in the EOs has already been proven. For instance, α-terpinene showed a remarkable inhibitory effect on *Salmonella pullorum* rather than *L. plantarum* [55]. Similarly, sabinene and γ-terpinene exhibited antibacterial activity on pathogenic bacteria such as *Streptococcus mutans* and very weak or no activity on *Lactobacillus* sp. (*L. acidophilus* and *L. casei*) [57]. Furthermore, (E)-caryophyllene showed inhibitory effects on *E. coli*, *Pseudomonas. aeruginosa*, *S. aureus* and *B. subtillis* [58]. The antibacterial activity of (E)-caryophyllene has been associated with its ability to alter the bacterial membrane permeability and provoke intracellular content leakage, resulting in cell death [59]. Likewise, a fraction of an EO containing decanal (73.4%) showed strong antibacterial activity on *E. coli* and *S. aureus* at 0.1 mg/mL (growth inhibition) and 0.2 mg/mL (killing) [60]. The combination of decanal and other compounds, such as γ-terpinene, was responsible for the antibacterial effects of a mandarin EO on *E. coli* and other strains [58]. The selective antibacterial performance of EODR could be more associated with the presence of α-terpinene, sabinene, and γ-terpinene, supported by the other mentioned minor compounds. Even though several compounds play a role in defining the biological properties of an EO, minor compounds may potentiate or modulate the activity of the major ones. Thus, minor and major compounds act in consortia to define the biological properties of an EO, such as its antibacterial activity [61]. Therefore, the selective antibacterial activity of EODR seems to result mainly from the minor compounds (detected in a higher amount than in EOP and EOMR), acting synergistically with its major compounds (α-pinene and β-pinene).

In our study, the EOs from propolis were also tested for their antioxidant activity in vitro. EOMR had the highest ABTS radical scavenging, FRAP, and phenolic content, followed by EODR and EOP. EODR exhibited a slightly higher antioxidant activity compared to EOP, with no significant difference in the ABTS radical scavenging capacity and phenolic content. These findings are comparable to the range of antioxidant activity commonly observed for the main bioactive products of propolis (ethanolic extract of propolis) [3]. Few studies have investigated the antioxidant activity of EOs from propolis. Previously, the EO from Brazilian brown propolis exhibited ABTS radical scavenging activity (IC_50_ 30.1 ± 8.11 µg/mL) [62]. Another study tested 25 EOs of propolis from different locations in China for their capacity to scavenge the DPPH radical. The authors showed that the EOs from temperate zones had the highest antioxidant activity [63]. Nevertheless, in our study, the EOs from propolis were not capable of scavenging the DPPH radical.

According to the literature, propolis contains up to 1% of EO, or rarely 2–3%, which is just a fraction of its complex composition with a low content of polyphenols—these are known for having antioxidant activity [4,51]. The low phenolic content in EOs can be explained by the modest volatility and partial water-solubility of phenolics that could be partly lost during hydrodistillation [64]. Nonetheless, some volatile compounds present in the EOs from propolis can also be responsible for their antioxidant activity [51]. In our study, the EOs from crude propolis and its residues were rich in terpenes, such as α-pinene and β-pinene, which have been considered responsible for the antioxidant activity of some EOs. However, the differential abundance of these major compounds in our samples and the lack of correlation with their antioxidant activity allows us to infer that the antioxidant capacity of the EOs is not likely to be related to these compounds. In contrast, the minor compounds (thuja-2,4(10)-diene, myrcene, *n*-octanal, ρ-cymene, acetophenone, and α-copaene), detected in a higher abundance in EOMR than EOP or EODR, may explain the antioxidant capacity of the former.

Some studies have found no association between the high abundance of α-pinene and β-pinene and the antioxidant activity of the EOs in which they occur. For instance, there was no direct relationship between the high abundance of α-pinene and the (negligible) antioxidant activity of rosemary EO [65]. Moreover, α-pinene and β-pinene showed poor antioxidant activity in ABTS, DPPH, and FRAP assays [58]. Monoterpene hydrocarbons such as α-pinene, β-pinene, sabinene, α-thujene, camphene, and limonene, except α-terpinene and γ-terpinene (which were detected in EOMR), have been shown to have weak or negligible antioxidant activity [66]. This supports our assumption that the six minor compounds present in EOMR play a key role in the antioxidant activity of this EO.

In previous studies, myrcene was shown to have antioxidant activity [67,68]. Compared to other compounds, myrcene exhibit a three-fold higher antioxidant activity than that of α-pinene [52]. In another study, myrcene and *ρ*-cymene were assayed individually and showed a considerable antioxidant activity—which was higher than that of α -pinene, β-pinene, limonene, and camphene [66]. Therefore, the occurrence of these minor compounds in EOMR and a possible synergistic effect between them could explain the high antioxidant performance exhibited by EOMR and determine its antioxidant mechanism.

The antioxidant mechanisms of EOs are associated with their capability to donate hydrogen or an electron to free radicals (ROS) as well as with their ability to detach unpaired electrons inside the aromatic structure to counter free radicals and protect biological molecules from oxidation [69,70]. The supplementation of pig diets with EOs contributed significantly to controlling body damages produced by the disruption in redox balance due to excess ROS species in the organism of pigs [69,71]. Thus, EOs such as propolis EOs, could be used to modulate the oxidative stress in pigs, and prevent DNA, membrane, protein, and lipid damage in their organism [71].

Although the biological properties of EOs extracted from propolis remain poorly explored [4], mounting evidence has suggested they can be a promising low-cost source of compounds with biological properties, particularly the EOs from propolis residues. These have potential applications as natural antimicrobial/antioxidant agents to replace antibiotics and synthetic antioxidant additives in pig production. Further research is highly encouraged to explore and reveal the sustainable applicability of EOs from propolis in the animal production industry [4].

## 5. Conclusions

Overall, the EOs from organic propolis residues (EOMR and EODR) exhibited either antioxidant or antibacterial activity higher than that of the EO from crude propolis (EOP). EODR was found to have mild antioxidant properties and showed selective antibacterial activity, that is, higher activity against the pathogenic bacterium model *E. coli* rather than the beneficial bacterium model *L. plantarum*. EOMR showed the highest antioxidant performance in terms of ABTS radical scavenging, FRAP, and phenolic content.

Each EO presented a different chemical profile, and some of their minor compounds were more pronouncedly detected in EOMR and EODR. These compounds are most likely to be associated with the biological activities observed for these EOs (antioxidant and selective antibacterial activity) rather than the major compounds, α-pinene and β-pinene.

Therefore, the EOs from organic propolis residues are a source of compounds with antibacterial/antioxidant properties with potential application as natural additives in pig feed. Our findings provide valuable information and insights into the current knowledge of the biological properties of EOs extracted from propolis.

## Figures and Tables

**Table 1:** Chemical composition (%) of the essential oils extracted from crude organic propolis and from its moist and dry residues.

Compound	RI_calc_	RI_lit_	EOP	EOMR	EODR
			%		
Hexanal	801	801	0.19	-	-
Tricyclene	922	926	1.54	-	-
α-Thujene	931	930	1.01	0.97	1.13
α-Pinene	938	939	66.48	42.17	16.25
Camphene	952	954	2.94	-	0.55
Thuja-2,4(10)-diene	958	960	2.04	5.02	4.67
Sabinene	977	975	0.45	-	0.50
β-Pinene	981	979	18.46	10.29	5.15
Myrcene	995	990	0.67	1.32	-
*n*-Octanal	1005	998	-	0.6	0.35
α-Terpinene	1020	1017	0.22	0.67	0.91
*p*-Cymene	1027	1024	0.81	2.29	2.07
Limonene	1032	1029	2.04	2.72	3.07
γ-Terpinene	1062	1059	0.26	0.71	0.99
Acetophenone	1068	1065	0.35	0.68	0.64
*n*-Nonanal	1105	1100	-	0.62	0.97
Ethyl benzoate	1172	1173	-	4.74	7.80
*n*-Decanal	1206	1201	-	0.52	1.32
α-Copaene	1377	1376	-	0.93	-
Ethyl decanoate	1395	1395	-	-	0.74
(E)-Caryophyllene	1426	1419	-	1.18	3.02
Zonarene	1529	1529	-	-	3.82
Total			97.46	75.43	53.95

RI_calc_: Retention index calculated; RI_lit_: Retention index from the literature; EOP: essential oil of propolis; EOMR: essential oil from the moist residue of propolis; EODR: essential oil from the dry residue of propolis. The column used was RTX5MS (RESTREK).

## Data Availability

Data present in this study are available on request from the corresponding author.

## Sample Availability

Samples of the essential oils tested in this study are available from the authors.

## References

[1] Kamatou G., Sandasi M., Tankeu S., Vuuren S.V., Viljoen A. (2019). Headspace analysis and characterisation of South African propolis volatile compounds using GCxGC–ToF–MS. Rev. Bras. Farmacogn..

[2] Tiveron A.P., Rosalen P.L., Ferreira A.G., Thomasi S.S., Massarioli A.P., Ikegaki M., Franchin M., Sartori A.G.D.O., Alencar S.M.D. (2020). Lignans as new chemical markers of a certified Brazilian organic propolis. Nat. Prod. Res..

[3] Tiveron A.P., Rosalen P.L., Franchin M., Lacerda R.C.C., Bueno-Silva B., Benso B., Denny C., Ikegaki M., De Alencar S.M. (2016). Chemical characterization and antioxidant, antimicrobial, and anti-inflammatory activities of South Brazilian organic propolis. PLoS ONE.

[4] Bankova V., Popova M., Trusheva B. (2014). Propolis volatile compounds: Chemical diversity and biological activity: A review. Chem. Cent. J..

[5] Bittencourt M.L.F., Ribeiro P.R., Franco R.L.P., Hilhorst H.W.M., de Castro R.D., Fernandez L.G. (2015). Metabolite profiling, antioxidant and antibacterial activities of Brazilian propolis: Use of correlation and multivariate analyses to identify potential bioactive compounds. Food Res. Int..

[6] Tobaldini-Valerio F.K., Bonfim-Mendonça P.S., Rosseto H.C., Bruschi M.L., Henriques M., Negri M., Silva S., Svidzinski T.I.E. (2016). Propolis: A potential natural product to fight Candida species infections. Future Microbiol..

[7] Bueno-Silva B., Marsola A., Ikegaki M., Alencar S.M., Rosalen P.L. (2017). The effect of seasons on Brazilian red propolis and its botanical source: Chemical composition and antibacterial activity. Nat. Prod. Res..

[8] Fernandes F.H., Guterres Z.D.R., Violante I.M.P., Lopes T.F.S., Garcez W.S., Garcez F.R. (2015). Evaluation of mutagenic and antimicrobial properties of brown propolis essential oil from the Brazilian Cerrado biome. Toxicol. Rep..

[9] De Francisco L., Pinto D., Rosseto H., Toledo L., Santos R., Tobaldini-Valério F., Svidzinski T., Bruschi M., Sarmento B., Oliveira M.B.P.P. (2018). Evaluation of radical scavenging activity, intestinal cell viability and antifungal activity of Brazilian propolis by-product. Food Res. Int..

[10] Amaral Duarte C.R., Eyng C., Murakami E., Vargas D., Nunes V. (2017). Propolis residue inclusion in the diet affects digestive enzyme activity in broiler chickens. Semin. Ciênc. Agrár..

[11] Cromwell G.L. (2002). Why and how antibiotics are used in swine production. Anim. Biotechnol..

[12] Teillant A., Brower C.H., Laxminarayan R. (2015). Economics of Antibiotic Growth Promoters in Livestock. Annu. Rev. Resour. Econ..

[13] Barton M.D. (2014). Impact of antibiotic use in the swine industry. Curr. Opin. Microbiol..

[14] EU Regulation (EC) (2003). No. 1831/2003 of the European Parliament and of the Council of 22 September 2003 on additives for use in animal nutrition. Off. J. Eur. Union.

[15] Food Safety Commission of Japan (2017). Antimicrobial-resistant Bacteria Arising from the Use of Colistin Sulfate in the Livestock (Antimicrobial-resistant Bacteria). Food Saf..

[16] Liu Y., Liu J.-H. (2018). Monitoring Colistin Resistance in Food Animals, An Urgent Threat. Expert Rev. Anti-Infect. Ther..

[17] Ministério da Agricultura, Pecuária e Abastecimento (MAPA) (2016). Instrução Normativa 45°, de 22 de novembro de 2016. Diário Off. União.

[18] Walsh T.R., Wu Y. (2016). China bans colistin as a feed additive for animals. Lancet Infect. Dis..

[19] Vanrolleghem W., Tanghe S., Verstringe S., Bruggeman G., Papadopoulos D., Trevisi P., Zentek J., Sarrazin S., Dewulf J. (2019). Potential dietary feed additives with antibacterial effects and their impact on performance of weaned piglets: A meta-analysis. Vet. J..

[20] Chowdhury S., Mandal G.P., Patra A.K., Kumar P., Samanta I., Pradhan S., Samanta A.K. (2018). Different essential oils in diets of broiler chickens: 2. Gut microbes and morphology, immune response, and some blood profile and antioxidant enzymes. Anim. Feed Sci. Technol..

[21] Omonijo F.A., Ni L., Gong J., Wang Q., Lahaye L., Yang C. (2018). Essential oils as alternatives to antibiotics in swine production. Anim. Nutr..

[22] Ambrosio C.M.S., Ikeda N.Y., Miano A.C., Saldaña E., Moreno A.M., Stashenko E., Contreras-Castillo C.J., Da Gloria E.M. (2019). Unraveling the selective antibacterial activity and chemical composition of citrus essential oils. Sci. Rep..

[23] Melliou E., Stratis E., Chinou I. (2007). Volatile constituents of propolis from various regions of Greece—Antimicrobial activity. Food Chem..

[24] Dos Reis A.S., Diedrich C., de Moura C., Pereira D., de Flório Almeida J., da Silva L.D., Plata-Oviedo M.S.V., Tavares R.A.W., Carpes S.T. (2017). Physico-chemical characteristics of microencapsulated propolis co-product extract and its effect on storage stability of burger meat during storage at −15 °C. LWT Food Sci. Technol..

[25] Santos E.L., da Silva F.C.B., da Conceição Pontes E., Lira R.C., Cavalcanti M.C.A. (2013). Resíduo do processamento do extrato de própolis vermelha em ração comercial para alevinos de Tilápia do Nilo (Oreochromis niloticus). Comun. Sci..

[26] Oliveira A.P., França H.S., Kuster R.M., Teixeira L.A., Rocha L.M. (2010). Chemical composition and antibacterial activity of Brazilian propolis essential oil. J. Venom. Anim. Toxins Incl. Trop. Dis..

[27] Adams R.P. (2007). Identification of Essential Oils by Gas Chromatography/Mass Spectrometry.

[28] CLSI (2012). Methods for Dilution Antimicrobial Susceptibility Tests for Bacteria That Grow Aerobically.

[29] Zwietering M.H., Jongenburger I., Rombouts F.M., Van’ A.K., Riet T. (1990). Modeling of the Bacterial Growth Curve. Appl. Environ. Microbiol..

[30] Al-Duais M., Müller L., Böhm V., Jetschke G. (2009). Antioxidant capacity and total phenolics of Cyphostemma digitatum before and after processing: Use of different assays. Eur. Food Res. Technol..

[31] Moraes-de-Souza R.A., Oldoni T.L.C., Regitano-D’Arce M.A.B., Alencar S.M. (2008). Antioxidant activity and phenolic composition of herbal infusions consumed in Brazil. CYTA J. Food.

[32] De Souza Silva A.P., Rosalen P.L., de Camargo A.C., Lazarini J.G., Rocha G., Shahidi F., Franchin M., de Alencar S.M. (2021). Inajá oil processing by-product: A novel source of bioactive catechins and procyanidins from a Brazilian native fruit. Food Res. Int..

[33] Singleton V.L., Orthofer R., Lamuela-Raventós R.M. (1999). Analysis of total phenols and other oxidation substrates and antioxidants by means of folin-ciocalteu reagent. Methods Enzymol..

[34] Sena-Lopes Â., Bezerra F.S.B., das Neves R.N., de Pinho R.B., de Oliveira Silva M.T., Savegnago L., Collares T., Seixas F., Begnini K., Henriques J.A.P. (2018). Chemical composition, immunostimulatory, cytotoxic and antiparasitic activities of the essential oil from Brazilian red propolis. PLoS ONE.

[35] Cunha I., Sawaya A.C., Caetano F.M., Shimizu M.T., Marcucci M.C., Drezza F.T., Povia G.S., Carvalho P.D.O. (2004). Factors that influence the yield and composition of Brazilian propolis extracts. J. Braz. Chem. Soc..

[36] Hossain M.B., Barry-Ryan C., Martin-Diana A.B., Brunton N.P. (2010). Effect of drying method on the antioxidant capacity of six Lamiaceae herbs. Food Chem..

[37] Nunes C.A., Guerreiro M.C. (2012). Characterization of Brazilian green propolis throughout the seasons by headspace GC/MS and ESI-MS. J. Sci. Food Agric..

[38] Kaškonienė V., Kaškonas P., Maruška A., Kubilienė L. (2014). Chemometric analysis of volatiles of propolis from different regions using static headspace GC-MS. Cent. Eur. J. Chem..

[39] Simionatto E., Facco J.T., Morel A.F., Giacomelli S.R., Linares C.E.B. (2012). Chiral analysis of monoterpenes in volatile oils from propolis. J. Chil. Chem. Soc..

[40] Si W., Gong J., Tsao R., Zhou T., Yu H., Poppe C., Johnson R., Du Z. (2006). Antimicrobial activity of essential oils and structurally related synthetic food additives towards selected pathogenic and beneficial gut bacteria. J. Appl. Microbiol..

[41] Ambrosio C.M.S., de Alencar S.M., de Sousa R.L.M., Moreno A.M., Da Gloria E.M. (2017). Antimicrobial activity of several essential oils on pathogenic and beneficial bacteria. Ind. Crops Prod..

[42] Ambrosio C.M.S., Contreras-Castillo C.J., Da Gloria E.M. (2020). In Vitro mechanism of antibacterial action of a citrus essential oil on an enterotoxigenic Escherichia coli and Lactobacillus rhamnosus. J. Appl. Microbiol..

[43] Liu Y., Espinosa C.D., Abelilla J.J., Casas G.A., Lagos L.V., Lee S.A., Kwon W.B., Mathai J.K., Navarro D.M.D.L., Jaworski N.W. (2018). Non-antibiotic feed additives in diets for pigs: A review. Anim. Nutr..

[44] Li S.Y., Ru Y.J., Liu M., Xu B., Péron A., Shi X.G. (2012). The effect of essential oils on performance, immunity and gut microbial population in weaner pigs. Livest. Sci..

[45] Fokt H., Pereira A., Ferreira A., Cunha A., Almeida Aguiar C. (2010). How do bees prevent hive infections? The antimicrobial properties of propolis. Curr. Res. Technol. Educ. Top. Appl. Microbiol. Microb. Biotechnol..

[46] Koru O., Toksoy F., Acikel C.H., Tunca Y.M., Baysallar M., Uskudar Guclu A., Akca E., Ozkok Tuylu A., Sorkun K., Tanyuksel M. (2007). In Vitro antimicrobial activity of propolis samples from different geographical origins against certain oral pathogens. Anaerobe.

[47] Ouwehand A.C., Tiihonen K., Kettunen H., Peuranen S., Schulze H., Rautonen N. (2010). In Vitro effects of essential oils on potential pathogens and beneficial members of the normal microbiota. Vet. Med..

[48] Gutierrez J., Barry-Ryan C., Bourke P. (2008). The antimicrobial efficacy of plant essential oil combinations and interactions with food ingredients. Int. J. Food Microbiol..

[49] Hayouni E.A., Bouix M., Abedrabba M., Leveau J.-Y., Hamdi M. (2008). Mechanism of action of Melaleuca armillaris (Sol. Ex Gaertu) Sm. essential oil on six LAB strains as assessed by multiparametric flow cytometry and automated microtiter-based assay. Food Chem..

[50] Przybyłek I., Karpiński T.M. (2019). Antibacterial properties of propolis. Molecules.

[51] Siheri W., Alenezi S., Tusiimire J., Watson D.G., Alvarez-Suarez J.M. (2017). The Chemical and Biological Properties of Propolis. Bee Products—Chemical and Biological Properties.

[52] Wang C.-Y., Chen Y.-W., Hou C.-Y. (2019). Antioxidant and antibacterial activity of seven predominant terpenoids. Int. J. Food Prop..

[53] Soković M., Glamočlija J., Marin P.D., Brkić D., Van Griensven L.J.L.D. (2010). Antibacterial effects of the essential oils of commonly consumed medicinal herbs using an in vitro model. Molecules.

[54] Burt S. (2004). Essential oils: Their antibacterial properties and potential applications in foods—A review. Int. J. Food Microbiol..

[55] Dorman H.J.D., Deans S.G. (2000). Antimicrobial agents from plants: Antibacterial activity of plant volatile oils. J. Appl. Microbiol..

[56] Guimarães A.C., Meireles L.M., Lemos M.F., Guimarães M.C.C., Endringer D.C., Fronza M., Scherer R. (2019). Antibacterial Activity of Terpenes and Terpenoids Present in Essential Oils. Molecules.

[57] Freires I.A., Denny C., Benso B., De Alencar S.M., Rosalen P.L. (2015). Antibacterial activity of essential oils and their isolated constituents against cariogenic bacteria: A systematic review. Molecules.

[58] Yi F., Jin R., Sun J., Ma B., Bao X. (2018). Evaluation of mechanical-pressed essential oil from Nanfeng mandarin (Citrus reticulata Blanco cv. Kinokuni) as a food preservative based on antimicrobial and antioxidant activities. LWT.

[59] Moo C.L., Yang S.K., Osman M.A., Yuswan M.H., Loh J.Y., Lim W.M., Lim S.H.E., Lai K.S. (2020). Antibacterial activity and mode of action of β-caryophyllene on Bacillus cereus. Pol. J. Microbiol..

[60] Liu K., Chen Q., Liu Y., Zhou X., Wang X. (2012). Isolation and Biological Activities of Decanal, Linalool, Valencene, and Octanal from Sweet Orange Oil. J. Food Sci..

[61] Bakkali F., Averbeck S., Averbeck D., Idaomar M. (2008). Biological effects of essential oils—A review. Food Chem. Toxicol..

[62] Lima V.H.M.D., Almeida K.D.C.R., Alves C.C.F., Rodrigues M.L., Crotti A.E.M., Souza J.M.D., Ribeiro A.B., Squarisi I.S., Tavares D.C., Martins C.H.G. (2019). Biological properties of volatile oil from Brazilian brown propolis. Rev. Bras. Farmacogn..

[63] Chi Y., Luo L., Cui M., Hao Y., Liu T., Huang X., Guo X. (2020). Chemical Composition and Antioxidant Activity of Essential Oil of Chinese Propolis. Chem. Biodivers..

[64] Amorati R., Foti M.C., Valgimigli L. (2013). Antioxidant activity of essential oils. J. Agric. Food Chem..

[65] Baratta Tiziana M., Dorman Damien H., Deans S.G., Figueiredo C.A., Barroso J.G., Ruberto G. (1998). Antimicrobial and antioxidant properties of some commercial essential oils. Flavour Fragr. J..

[66] Ruberto G., Baratta M.T. (2000). Antioxidant activity of selected essential oil components in two lipid model systems. Food Chem..

[67] Ojeda-Sana A.M., van Baren C.M., Elechosa M.A., Juárez M.A., Moreno S. (2013). New insights into antibacterial and antioxidant activities of rosemary essential oils and their main components. Food Control.

[68] Xanthis V., Fitsiou E., Voulgaridou G.P., Bogadakis A., Chlichlia K., Galanis A., Pappa A. (2021). Antioxidant and cytoprotective potential of the essential oil pistacia lentiscus var. Chia and its major components myrcene and α-pinene. Antioxidants.

[69] Simitzis P.E. (2017). Enrichment of Animal Diets with Essential Oils—A Great Perspective on Improving Animal Performance and Quality Characteristics of the Derived Products. Medicines.

[70] Giannenas I., Bonos E., Christaki E., Florou-paneri P. (2013). Essential Oils and their Application in Animal Nutrition. Med. Aromat. Plants.

[71] Hao Y., Xing M., Gu X. (2021). Research Progress on Oxidative Stress and Its Nutritional Regulation Strategies in Pigs. Animals.

